# Strong association between hla-a*02:06 and acetaminophen-related stevens-johnson syndrome with severe mucosal involvements in the Japanese

**DOI:** 10.1186/2045-7022-4-S3-P11

**Published:** 2014-07-18

**Authors:** Mayumi Ueta, Chie Sotozono, Hiromi Sawai, Katsushi Tokunaga, Shigeru Kinoshita

**Affiliations:** 1 Kyoto Prefectural University of Medicine and Doshisha University, Japan; 2 Kyoto Prefectural University of Medicine, Department of Ophthalmology, Japan; 3 Graduate School of Medicine, University of Tokyo, Department of Human Genetics, Japan

## Background

Stevens-Johnson syndrome (SJS) and its severe variant, toxic epidermal necrolysis (TEN), are acute inflammatory vesiculobullous reactions of the skin and mucous membranes of, for example, the ocular surface, oral cavity, and genitals. They are rare but often associated with inciting drugs. Cold medicines including non-steroidal anti-inflammatory drugs (NSAIDs) and multi-ingredient cold medications are reported to be important inciting drugs. We investigated the association between HLA genotypes and cold medicine-related SJS/TEN (CM-SJS/TEN) with severe mucosal involvement such as severe ocular surface complications (SOC). We also investigated the association between HLA genotypes and acetaminophen-related SJS/TEN in patients with CM-SJS/TEN with SOC.

## Methods

Japanese acetaminophen-related SJS/TEN patients consist of 59 patients, they were among the 131 CM-SJS/TEN with SOC. While the specific drugs were not named by all 131 patients, 59 (45.0%) were known to have taken medicines containing acetaminophen. The diagnosis of SJS/TEN with SOC was based on a confirmed history of acute-onset high fever, serious mucocutaneous illness with skin eruptions, and the involvement of at least 2 mucosal sites including the oral cavity and ocular surface. We analyzed HLA-A, -B, and -C of 131 CM-SJS/TEN patients and 419 Japanese controls.

## Results

HLA-A*02:06 was strongly associated with CM-SJS/TEN with SOC (carrier frequency: p=2.8X10-16, Pc=4.8X10-15, OR=5.7), and the odds ratio was highest for acetaminophen-related SJS/TEN with SOC (carrier frequency: p=5.0X10-13, Pc=8.5X10-12, OR=7.0).

## Conclusion

Elucidating the predisposition to drug-induced severe cutaneous adverse reactions might make it possible to prevent drug-induced severe cutaneous adverse reactions with avoiding the risk drug from the person having the predisposition.

